# Increase in circulating GLP-1 following low FODMAP diet in irritable bowel syndrome patients

**DOI:** 10.3389/fnut.2025.1615671

**Published:** 2025-08-13

**Authors:** Umael Khan, Ingeborg Brønstad, Eline Margrete Randulff Hillestad, Elisabeth K. Steinsvik, Trygve Hausken, Birgitte Berentsen, Gülen Arslan Lied

**Affiliations:** ^1^Section of Gastroenterology, Department of Medicine, Haukeland University Hospital, Bergen, Norway; ^2^Department of Medicine, National Centre for Ultrasound in Gastroenterology, Haukeland University Hospital, Bergen, Norway; ^3^National Centre for Functional Gastrointestinal Disorders, Haukeland University Hospital, Bergen, Norway; ^4^Department of Clinical Medicine, Centre for Nutrition, University of Bergen, Bergen, Norway

**Keywords:** FODMAP, IBS, GLP-1, incretins, gut microbiota, brain-gut

## Abstract

**Background:**

Fermentable oligosaccharides, disaccharides, monosaccharides and polyols (FODMAP) as well as glucagon-like peptide-1 (GLP-1) have independently been implicated in irritable bowel syndrome (IBS) pathophysiology. However, there is a lack of studies that assess how low FODMAP diet affects circulating GLP-1 levels in IBS patients.

**Methods:**

Thirty patients with either diarrhea or mixed type IBS were recruited and undertook low FODMAP diet for 12 weeks. Plasma GLP-1 levels, IBS Severity Scoring System (IBS-SSS), body weight and FODMAP intake were assessed before and after the 12-week dietary intervention.

**Key results:**

Following a low FODMAP diet, average IBS-SSS and body weight were reduced (*p* < 0.01) and plasma GLP-1 level was increased (*p* = 0.027).

**Conclusion:**

Our study indicates that a 12-week low FODMAP diet may increase plasma glucagon-like peptide-1 levels in IBS patients. The underlying mechanism for this increase remains to be understood.

## 1 Introduction

Irritable bowel syndrome (IBS), as classified by the Rome III and IV criteria, is one of the most common gastrointestinal conditions and causes a substantial reduction in quality of life (1). Depending on the predominant symptoms it is divided into IBS-diarrhea (IBS-D), IBS-constipation (IBS-C), IBS-mixed (IBS-M) or IBS-unspecified (IBS-U). The prevalence is 5–10% in most parts of the world, with the caveat of a shortage of studies in Africa, Eastern Europe and the Middle East (2). The etiology is multifactorial and incompletely understood (2). The enteroendocrine system, especially glucagon like peptide 1 (GLP-1) has been implicated in IBS pathophysiology, and dietary intake of fermentable oligosaccharides, disaccharides, monosaccharides and polyols (FODMAP) frequently exacerbates IBS symptoms (3, 4). While both low FODMAP diet (LFD) and circulating GLP-1 have been assessed in the context of IBS, there is a lack of studies on how they affect each other.

With regards to the enteroendocrine system, previous studies have shown changes in concentrations of enteroendocrine stem cells in IBS patients (5), as well as changes in circulating GLP-1 (6, 7). Furthermore, GLP-1 analogs have shown therapeutic potential in IBS patients (8–10). From the dietary perspective, undigested food particles in the colon can be rapidly fermented by the colonic bacteria, which in turn can contribute to abdominal symptoms and discomfort (4). LFD has shown substantial symptomatic improvement in IBS patients, in particular IBS-D and IBS-M patients (11–15).

Understanding the connection between LFD and GLP-1 can further advance our understanding of IBS as well as improve treatment. The aim of this study is therefore to assess how LFD affects circulating GLP-1 in IBS-D and IBS-M patients. This study is part of the interdisciplinary Bergen Brain-Gut project (16).

## 2 Methods

### 2.1 Study participants

As part of the Bergen Brain-Gut project (16), 30 patients with IBS were recruited through social media, advertisements in the local newspaper as well as through the IBS outpatient clinic at Haukeland University Hospital between May 2019 and February 2021. Details regarding patient recruitment can found in the Bergen Brain-Gut 2protocol (16). All subjects were provided with oral and written information, and written consent was obtained. The study was conducted in compliance with the Declaration of Helsinki and the protocol was approved by The Regional Committee for Medical and Health Research Ethics Southeast in Norway (REK2015-1621).

The inclusion criteria were otherwise healthy patients in the age range 18–65 years that fulfilled the Rome IV criteria for IBS (17) and also had an IBS Severity Scoring System (IBS-SSS) score > 175, corresponding to moderate to severe IBS. Exclusion criteria included any pharmacological treatment of the gastrointestinal tract, systemic antibiotics within the last 3 months, probiotics or low-FODMAP-diet within the last 3 weeks, vegan or vegetarian diet, regular use of analgesics, pregnancy, prior gastrointestinal surgery apart from appendectomy (16). Thirty patients with either IBS-M or IBS-D went through a 12-week strict LFD dietary intervention where intake of all FODMAP groups were avoided. The diet was guided by a registered dietitian with monthly follow-ups for assessment of compliance and safety. Further details regarding study design, including power calculations, are provided in the Bergen Brain-Gut project protocol (16). FODMAP intake was assessed by registering 3 days of their normal diet (two weekdays and 1 day during the weekend) and plotted into Monash FODMAP Calculator (Monash University, Melbourne, Australia) at baseline, week 4 and week 12. IBS symptoms were evaluated using the IBS-SSS at baseline and after 12 weeks of on the LFD, IBS-SSS was chosen as the primary measure of symptomatic response due to its widespread application in IBS research (18). In addition, this was supplemented by Gastrointestinal Symptom Rating Scale (GSRS)-IBS before and 12 weeks after LFD. Body weight was recorded at baseline and after 12 weeks of LFD.

### 2.2 Blood sampling and GLP-1 analyses

Blood samples after an overnight fast were acquired to determine GLP-1 concentration at baseline and after a 12-week strict LFD: Blood was drawn into pre-cooled 1 mL VACUETTE®EDTA-K2 blood collecting tubes (cat # G454052) with 10 μL dipeptidyl peptidase-4 inhibitor (DPP4-010; DRG Diagnostics, Marburg, Germany) added prior to sampling. Blood samples were centrifuged at 1,800 × g at −4°C for 10 min within 20 min after sampling. Plasma for GLP-1 analysis was then aliquoted and stored at −80°C. The GLP-1 analyses were performed using an ELISA (enzyme-linked immunosorbent assay) kit (Millipore, GLP-1 (7–36). Active ELISA kit, catalog # EGLP-35 K, Merck KGaA, Darmstadt, Germany). The ELISA fluorescence readings of 355 nm/460 nm (excitation/emission) were carried out using a SPECTRA MAX GEMINI EM microplate reader (Molecular Devices, Sunnyvale, CA, United States) and concentrations were calculated by the SoftMaxPro Software version 7.1 (Molecular Devices, Sunnyvale, CA, United States) using linear curve.

### 2.3 Statistical analyses

The statistical analyses were carried out with the SPSS statistical package version 25 (SPSS Inc., IBM Corp., Armonk, NY, United States). We used the Shapiro–Wilk test to assess distribution, and we also screened for outliers in our data sets. Baseline values were compared with post LFD values using either paired t-tests or Wilcoxin signed-rank depending on the distribution of the data. In case of dropout, an intention-to-treat approach was applied in order to prevent risk of bias as long as the assessed variable itself was available. Correlations between change in GLP-1 and change in bodyweight as well as change in IBS-SSS were assessed using visual inspection of scatterplots and by assessment of either Pearson’s correlation, or if assumptions of normality and linearity were violated, by Spearman’s rank-order correlation. Correlation between change in GSRS sub scores and change in IBS-SSS was also assessed using the same methods.

## 3 Results

Patient characteristics acquired at baseline are presented in Table 1. Whereas GLP-1 levels were acquired from all 30 individuals, there was some dropout in the secondary variables due to COVID-19 pandemic restrictions. Hence, *n* = 29 for IBS-SSS score, *n* = 23 for GSRS, *n* = 24 for change in bodyweight and *n* = 15 for change in total FODMAP intake from baseline to week 12.

**Table 1:** Patient characteristics.

Age (years)	38 ± 12
Gender (number male/number patients)	9/30
IBS subtype (number IBS-D/IBS-M)	11/19
Bodyweight at baseline (kg)	78.4 ± 18.4

Continuous variables provided as mean ± standard deviation. IBS, irritable bowel syndrome; IBS-D, IBS diarrhea predominant; IBS-M, IBS mixed type; LFD; Low fermentable oligosaccharides, disaccharides, monosaccharides, and polyols diet.

**Figure 1:**
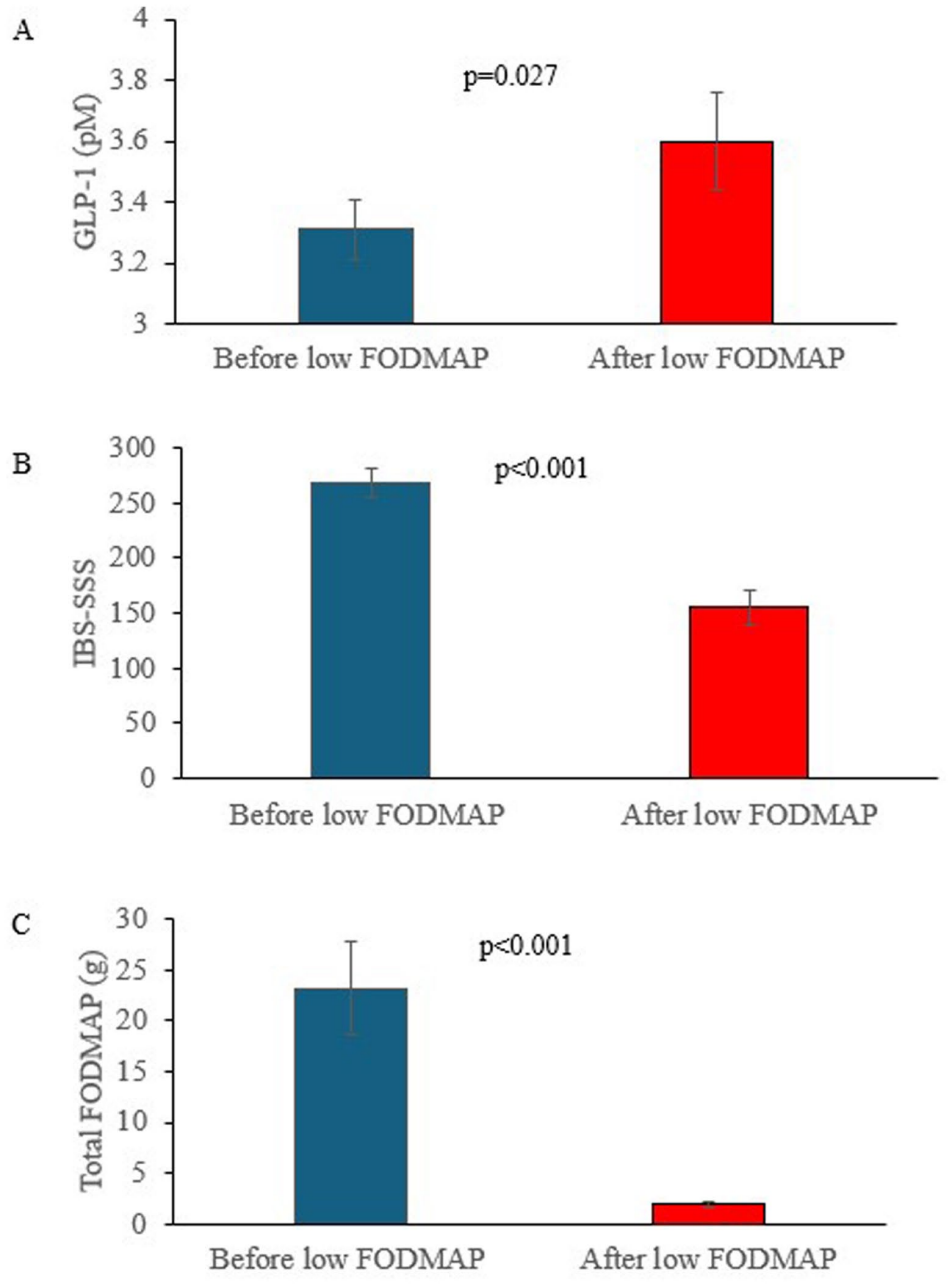
**(A)** Increase in glucagon like peptide 1 (GLP-1) expressed as picomoles (pM) following low fermentable oligosaccharides, disaccharides, monosaccharides and polyols diet (LFD), *n* = 30. **(B)** Reduction in irritable bowel syndrome severity scoring symptom (IBS-SSS) following LFD, *n* = 29. **(C)** Reduction in fermentable oligosaccharides, disaccharides, monosaccharides and polyols (FODMAP) following the LFD, *n* = 15.

Average GLP-1 (mean ± standard deviation) levels increased from 3.3 pM ± 0.5 pM to 3.6 pM ± 0.9 pM following the dietary intervention, *p* = 0.027 (Figure 1A). IBS-SSS (mean ± standard deviation) was substantially reduced from 269.8 ± 67.1 to 155.3 ± 93.1, *p* < 0.001 (Figure 1B). Along the same lines, there was a statistically significant reduction in GSRS scores for pain, bloating and diarrhea (values presented in Table 2). Average weight (mean ± standard deviation) was reduced from 78.4 kg ± 18.4 kg to 76.6 kg ± 17.0 kg. Total FODMAP intake (mean ± standard deviation) was also substantially reduced from 24.8 g ± 23.6 g at baseline to 2.1 g ± 1.5 g at week 12, *p* < 0.001 (Figure 1C). The reduction in FODMAP from baseline to week 12 was only acquired from 15 patients due to COVID restrictions. However, if one includes dietary records from week 4, 25/30 patients delivered FODMAP intake records at either week 4 and/or week 12. Average FODMAP intake at week 4 (mean ± standard deviation) was 1.6 g ± 1.6 g.

**Table 2:** Change in Gastrointestinal Symptom Rating Scale.

	Before LFD	After LFD	*p* value
GSRS pain	7.7 ± 1.6	6.2 ± 2.1	0.004
GSRS bloating	13.8 ± 2.6	10.1 ± 2.9	<0.001
GSRS constipation	5.5 ± 3.1	4.9 ± 2.7	0.224
GSRS diarrhea	14.5 ± 4.5	10.8 ± 5.1	<0.001
GSRS satiety	4.8 ± 2.5	4.1 ± 1.9	0.197

GSRS-IBS, Gastrointestinal Symptom Rating Scale; LFD, low fermentable oligosaccharides, disaccharides, monosaccharides, and polyols diet.

**Figure 2:**
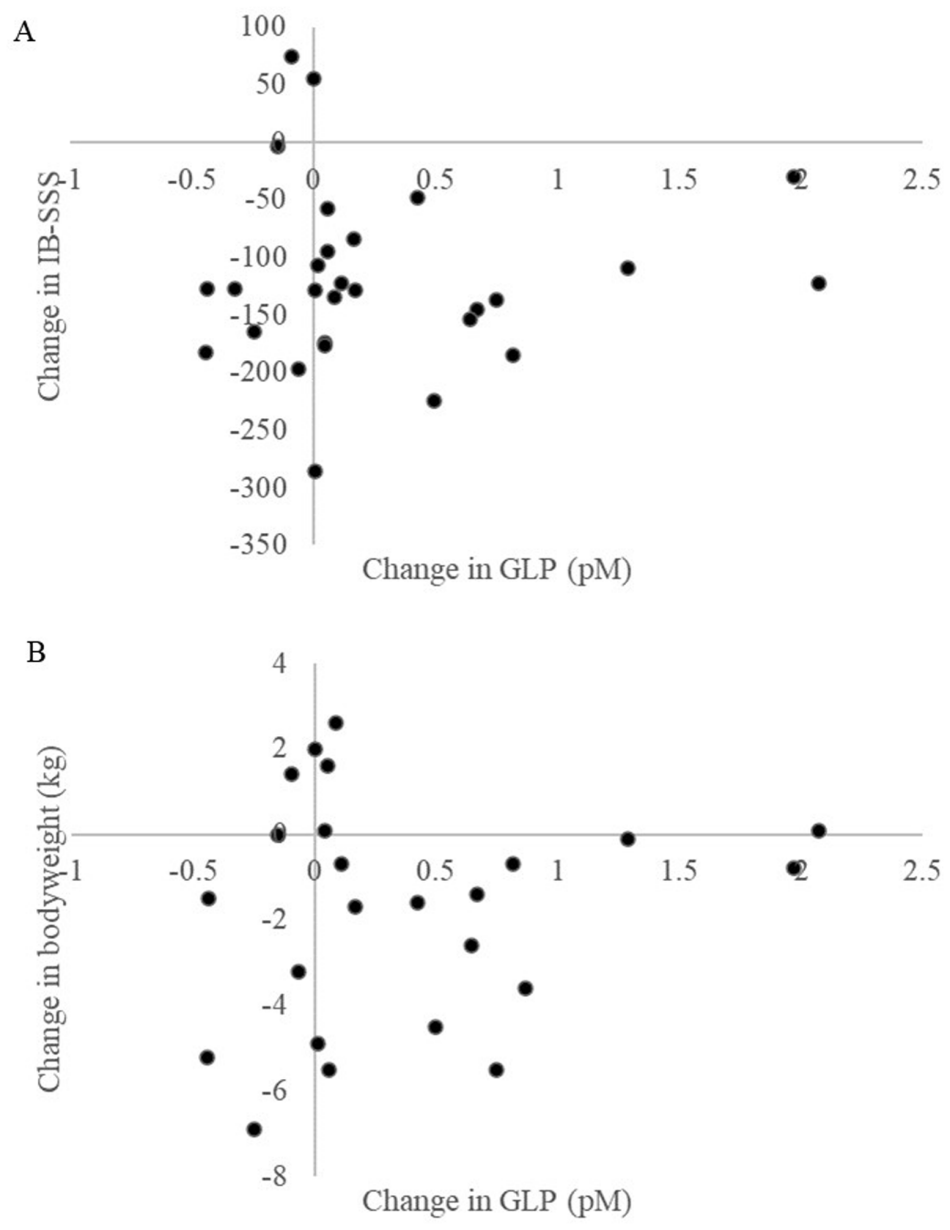
**(A)** Scatterplot showing the change in glucagon like peptide 1 (GLP-1) expressed as picomoles (pM) with corresponding change in irritable bowel syndrome severity scoring system (IBS-SSS). Correlation coefficient = 0.02, *p* = 0.93. **(B)** Scatterplot showing change in glucagon like peptide 1 (GLP-1) expressed as picomoles (pM) with corresponding change in bodyweight (kg). Correlation coefficient = 0.07, *p* = 0.76.

We did not see any correlation between changes in plasma GLP-1 levels and changes in IBS-SSS (correlation coefficient = 0.02, *p* = 0.93) or changes in bodyweight (Correlation coefficient = 0.07, *p* = 0.76) (Figure 2). Furthermore, there was no significant correlation between change in the GSRS sub scores and change in GLP-1 levels (scatterplots, correlation coefficients and *p* values for each sub score is presented in Supplementary material).

## 4 Discussion

The etiology of IBS is not fully understood, although biopsychosocial models such as the brain-gut axis are currently used to highlight the multifactorial pathophysiology of this condition (2, 16, 19, 20). To the best of our knowledge, this is the first study to assess the effect of LFD on circulating GLP-1. Our main finding is that LFD is associated with an increase in circulating GLP-1 levels in IBS patients. However, the underlying mechanisms remain to be identified.

While there was an average reduction in bodyweight, a previous study on nutritional safety following LFD did not find any clinically meaningful changes in in macro- or micronutrient intake in spite of weight loss, indicating its relative safety (21). There was no clinically significant correlation between change in GLP-1 and change in bodyweight. Some studies indicate that GLP-1 levels are positively correlated with body mass index (BMI), and weight loss reduces circulating levels of GLP-1 (22, 23). The lack of correlation between change in GLP-1 levels and change in bodyweight in our study could indicate that change in GLP-1 levels following LFD were not solely due to change in body weight. With regards to symptom improvement, while there was a substantial reduction in IBS-SSS (as well as GSRS sub scores for diarrhea, bloating and pain), change in IBS-SSS was not correlated with changes in GLP-1. A direct association between symptomatic improvement and changes in GLP-1 levels was therefore not seen in this study. While the correlation analyses were hampered by drop-out and thereby a low number of subjects (n = 29 for IBS-SSS, n = 24 for change in bodyweight), the scatterplots nonetheless indicate that a strong correlation is unlikely.

Although the mechanisms behind the increase in GLP-1 following LFD remain unknown, hypotheses can be drawn from previous studies. It is already known that food intake affects gut hormones, possibly through chemosensory mechanisms in enteroendocrine cells (24). L-cells in the colon are exposed to microbial metabolites such as short chain fatty acids and secondary bile acids (25). Dietary changes such as LFD could affect the exposure of L-cells to these metabolites, which in turn could affect the production of GLP-1.

Several pathophysiological components of IBS are associated with both FODMAP intake and GLP-1. These components could mediate the connection between LFD and GLP-1 in IBS patients. Compromised intestinal epithelial barrier likely plays a role in IBS and could be one such component (26, 27). A previous study has shown that exogenous administration of GLP-1 could improve intestinal barrier function (28). At the same time, FODMAP intake also affects intestinal barrier function (29). It is therefore possible that intestinal epithelial barrier mediates the relationship between LFD and GLP-1.

Gut motility is another important component of IBS. Like the intestinal epithelial barrier it is affected by both FODMAPs and GLP-1 (30, 31). GLP-1 is well known for its effect in reducing gut motility in the stomach and small intestine (32, 33). However, its effect on the colon is more controversial with discrepant findings regarding its effect on colonic transit time (34, 35). Previous studies also show discrepant results regarding the level of circulating GLP-1 in IBS patients (6–10). Treatment with GLP-1 analogs leads to symptomatic improvement in IBS patients (8, 10). At the same time, both diarrhea and constipation are common side effects of GLP-1 analogs (36, 37), indicating a complex mechanism at play. Dietary composition also likely plays a part in gut motility, as indicated by differential gut manometry responses to FODMAP infusions in IBS patients compared to healthy controls (38). Hence, it is possible that the association between LFD and GLP-1 levels is also partly mediated by gut motility. Beyond gut motility, visceral hypersensitivity is also an important aspect of the brain-gut axis in IBS. The mechanism is not fully understood, but inflammation, abnormality of the gut’s mechanoreceptors and emotional states such as hypervigilance and stress likely play a role (39). Studies show that GLP-1 analogs as well as LFD affect visceral hypersensitivity (40, 41).

A better understanding of both GLP-1 and LFD as well as their connection has important therapeutic implications. LFD is a well-established treatment in IBS, especially IBS-D and IBS-M patients (11–15). GLP-1 analogs such as ROSE-010 have also shown therapeutic potential. A randomized clinical trial has shown ROSE-010 to provide substantial pain relief in IBS patients (8). The effects of GLP-1 analogs in IBS could also go beyond pain relief; Camillieri et al. found that ROSE-010 had a significant effect on gastrointestinal motility, with effects varying according to location within the gastrointestinal tract (9). Interestingly, while ROSE-010 effects seem to be dose dependent in previous studies, we did not find a correlation between the amount of change in GLP-1 and amount of IBS-SSS reduction (9, 10). Whether this is due to the limited increase in GLP-1 from 3.3 pM ± 0.5 pM (before LFD) to 3.6 pM ± 0.9 pM (after LFD) should be investigated in future studies. Understanding the association between GLP-1 and LFD could help further develop treatment for IBS.

This is the first study that examines the effect of LFD on circulating GLP-1 in IBS patients. It is a small single-arm interventional study that focuses on a single research question, namely whether LFD affects circulating GLP-1 levels in IBS-M/D. It should be noted that this is a single-center study that only examines a single dietary intervention. Furthermore, this was a study on IBS patients before and after the LFD intervention; therefore, there was no healthy control group. Hence, this study does not reveal whether GLP-1 levels are different in IBS patients compared to healthy controls, as that was not the aim of this study. Rather, it only shows that GLP-1 levels rise following LFD in IBS-M/D patients. Whereas previous studies have compared circulating GLP-1 in IBS and healthy controls, these studies have either focused on IBS-C or have been conducted in animal models (6, 7). Our study assessed IBS D/M patients rather than IBS-C as they were deemed most likely to respond to a LFD (42). Further studies are therefore recommended to compare circulating GLP-1 levels in different IBS subgroups and healthy controls. Such studies could also help further our understanding of the pathophysiological basis of the change in GLP-1 levels seen in this study.

Although statistically significant changes in GLP-1 levels were obtained, the small sample size of 30 patients warrants further, larger studies to verify our findings. This study could therefore be interpreted as a pilot study. While GLP-1 was obtained in all 30 patients, there were some drop-out due to COVID restrictions for the main secondary variables of total changes in bodyweight, IBS-SSS and total FODMAP intake. Nonetheless, statistically significant changes in the abovementioned variables were seen. This study only assessed one dietary intervention, namely LFD. More studies are recommended to assess how other dietary treatments affect enteroendocrine hormones (43). For instance, a prior study has examined the effect of a starch and sucrose reduced diet and did not find a significant change in GLP-1 (44). Given the multifactorial nature of IBS, the results of this study should be interpreted and extrapolated with caution.

The substantial reduction in total FODMAP intake indicates good compliance with the LFD. Although only 15/30 patients delivered complete FODMAP dietary records at baseline and week 12, if one assesses dietary intake at week 4 as well, 25/30 patients delivered dietary records of total FODMAP intake at week 4 and/or week 12. The average FODMAP intake was low at both weeks 4 and 12, indicating good adherence to the diet. Nonetheless, the incomplete dietary record is an important limitation.

## 5 Conclusion

This study is the first to assess change in plasma GLP-1 levels following LFD in IBS patients. An increase in GLP-1 levels as well as a reduction in IBS-SSS was found following LFD. However, no direct correlation between the amount of change in IBS-SSS and GLP-1 was found. Given the therapeutic potential of both dietary interventions as well as GLP-1 analogs, understanding how dietary interventions affect circulating GLP-1 can help further improve IBS treatment. However, future studies that assess the underlying mechanisms behind GLP-1 changes are recommended in order to better understand this mechanism.

## Data Availability

The raw data supporting the conclusions of this article will be made available by the authors, without undue reservation.
