# Supplementary material for: Increase in circulating GLP-1 following low FODMAP diet in irritable bowel syndrome patients
Source: Front Nutr. 2025 Aug 13;12:1615671. doi: 10.3389/fnut.2025.1615671 (PMC12380553; doi:10.3389/fnut.2025.1615671)
Supplement: Supplementary file 1 [file Table_1.docx]

Supplementary material:

Correlations between change in Gastrointestinal Symptom Rating Scale (GSRS) subscores and change in glucagon like peptide 1 (GLP-1) following a low fermentable oligosaccharides, disaccharides, monosaccharides and polyols (FODMAP) diet


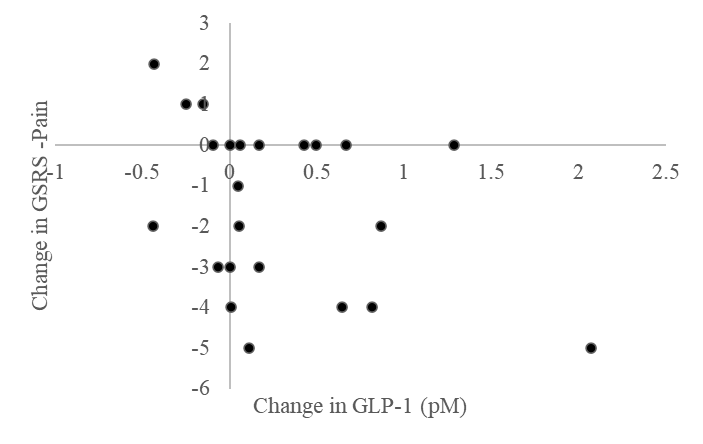


Correlation coefficient -0.38, p=0.08


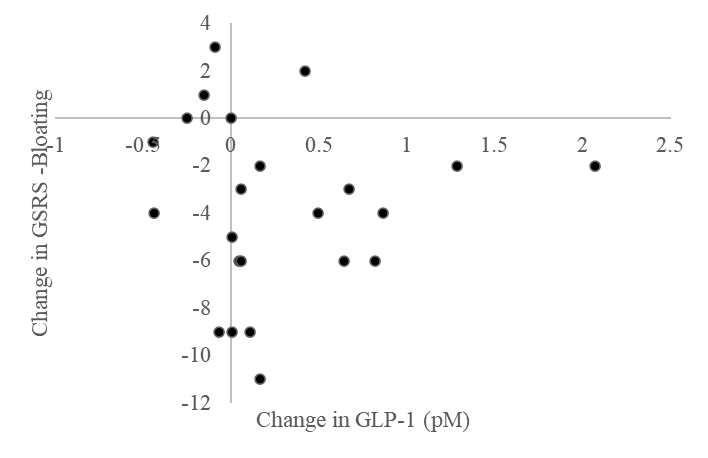


Correlation coefficient -0.18, p=0.42


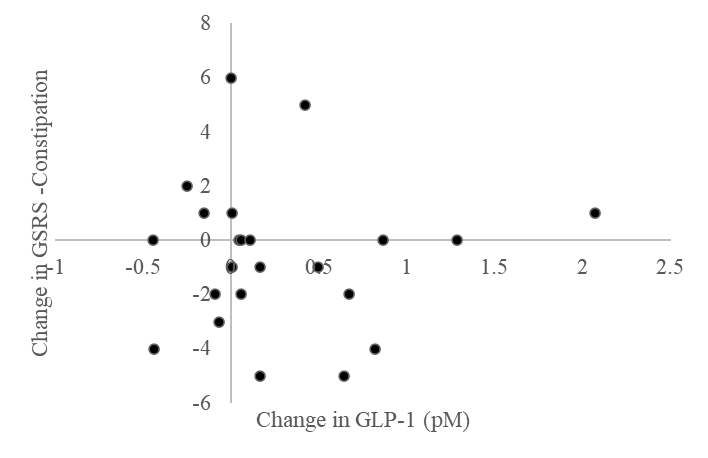


Correlation coefficient -0.12, p=0.60

Correlation coefficient 0.17, p=0.45


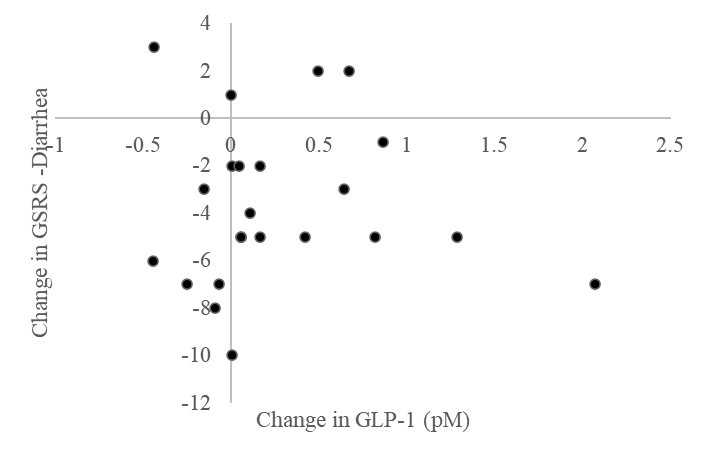


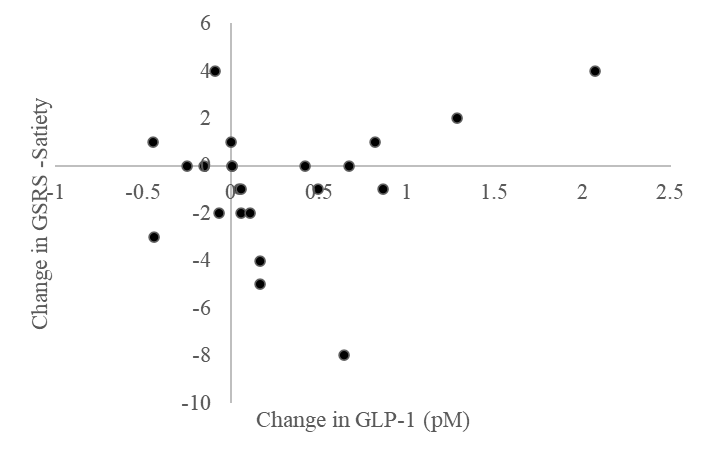


Correlation coefficient 0.02, p=0.91
